# The pretreatment Controlling Nutritional Status (CONUT) score is an independent prognostic factor in patients undergoing resection for colorectal cancer

**DOI:** 10.1038/s41598-020-70252-2

**Published:** 2020-08-06

**Authors:** Tamuro Hayama, Tsuyoshi Ozawa, Yuka Okada, Mitsuo Tsukamoto, Yoshihisa Fukushima, Ryu Shimada, Keijiro Nozawa, Keiji Matsuda, Shoichi Fujii, Yojiro Hashiguchi

**Affiliations:** 1 Department of Surgery, Teikyo University School of Medicine, 2-11-1 Kaga, Itabashi-ku, Tokyo, 173-8605 Japan; 2 Department of Surgery, Koga Hospital, Yaizu, Japan

**Keywords:** Cancer, Surgical oncology

## Abstract

The Controlling Nutritional Status (CONUT) score is a marker of nutrition and is associated with poor survival in various kinds of cancers. However, no reports have yet compared risk factors for colorectal cancer recurrence using a nutritional index. We assessed the predictive value of the CONUT score compared with the modified Glasgow Prognostic Score (mGPS) and Prognostic Nutritional Index (PNI) in colorectal cancer (CRC) patients. We performed a retrospective cohort study of the medical records of 336 consecutive patients with stage I-I I I CRC who underwent curative resection at a single institution in 2012–2017. Univariate and multivariate analyses were conducted to identify prognostic factors associated with relapse-free survival (RFS) and overall survival (OS). The low CONUT score group exhibited higher RFS and longer OS compared to the high CONUT score group (82.2% vs. 63.3%, *p* = 0.002 and 95.5% and 86.2%, *p* = 0.005, respectively). The Akaike’s information criterion values of each index for RFS and OS were superior in CONUT score (723.71 and 315.46, respectively) compared to those of PNI (726.95 and 316.52) and mGPS (728.15 and 318.07, respectively). The CONUT score was found to be a good predictor of RFS and OS in patients with resectable CRC.

## Introduction

Colorectal cancer (CRC), one of the most common and aggressive malignancies, is the fourth-leading cause of cancer-related death worldwide^1^. The Controlling Nutritional Status (CONUT) score—which is comprised of the serum values of albumin (ALB) , total lymphocyte count (TLC), and total cholesterol—is considered a potential predictor of poor outcomes in several types of malignances, including colorectal cancer^2,3^. Two nutritional indicators, i.e., the modified Glasgow Prognostic Score (mGPS) and the Prognostic Nutritional Index (PNI), have been widely established predictors of the prognoses of patients with cancer^4–6^.

We, clinicians, need tools that are simpler and more straightforward than complex diagnostic and prognostic tools. A variety of laboratory biomarkers have been developed that use the systemic inflammation or nutritonal status of patients as a simple tool. These biomarkers have been demonstrated to predict the prognosis in various cancers, and several studies have demonstrated that these indicators are predictive of the prognosis of patients with CRC^2,7,8^. However, to the best of our knowledge, there is no report on which prognostic indicators are the most useful for CRC patients after curative surgery. We conducted the present study to determine the utility of these predictive indicators, based on nutritional factors, in CRC patients after their curative surgery.

## Results

### Patient characteristics

The median age of the 301 patients was 67 (range, 22–93) years; 180 (60.1%) patients were male and 121 (39.9%) were female. Primary tumors were located in the right of the colon in 91 cases (30.2%) and in the left side of the colon or rectum in 210 (69.8%). Histologically, 278 tumors (89.0%) were moderately or well differentiated, and the other 33 (11.0%) were not (Table 1). Seventy-seven (25.6%) early complications were noted; these comprised surgical site infections (n = 20), ileus (n = 10), anastomosis leakage (n = 8), delirium (n = 7), respiratory infections (n = 6), urinary disturbances (n = 5), urinary infections (n = 5), colitis (n = 3), hemorrhage (n = 6), and complications (n = 8).

**Table 1:** Clinicopathological features of the stage II colorectal cancer patients who underwent curative tumor resection.

	n = 301 (%)			
Gender	Male	180 (60.8)	Female	121 (40.2)
Age (years), median	Average	67.74		
Location of cancer	Right side	91 (30.2)	Left side	210 (69.8)
Histology	tub1, tub2	268 (89.0)	Others	33 (11)
Vascular invation	(+)	90 (30.2)	(−)	201(66.7)
Lymph invation	(+)	142 (47.2)	(−)	159 (52.8)
pT category (T1,2:T3≦)	T1,2	93 (30.9)	T3 ≤	208 (69.1)
N category	(+)	189 (62.8)	(−)	112 (37.2)
Preoperative CEA levels	Normal	201 (66.8)	Higher	100 (33.2)
Preoperative CA 19-9 levels	Normal	250 (83.1)	Higher	51 (16.9)
Early complications	(+)	77 (25.6)	(−)	224 (74.4)
CONUT score	3>	195 (64.8)	3 ≤	106 (35.2)

### Determination of cut-off values

The ROC curve analysis results indicated that the most appropriate cutoff value for the CONUT score was 3. All patients were categorized into the high CONUT score group (score ≥ 3; n = 106, 35.2%) or low CONUT score group (score = 0 or 1 or 2; n = 195, 64.8%).

### ALB, TLC, cholesterol scattergraph by CONUT score

**Figure 1:**
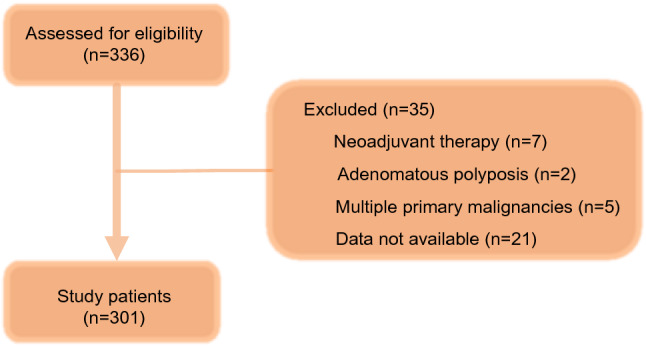
Flow diagram of the cases analyzed in this study.

The mean values of the low CONUT score group were as follows. ALB: 4.1, TLC: 1,641, and cholesterol: 195, and those of the high CONUT score group were 3.4, 1,101, and 159 (Fig. 1). All items were significantly different between the two groups. None of the patients in the low-cholesterol group was taking cholesterol medication.

### Prognostic factors

We used a univariate analysis and the Cox regression model to identify risk factors for recurrence after surgery. It is difficult to evaluate the nutritional indices together as prognostic factors because there is strong confounding among them. We examined each nutritional index and clinicopathological factors. A CONUT score ≥ 3 lymphatic invasion, vascular invasion, pT category, preoperative CEA level, and early complications were significantly associated with poor RFS in the univariate survival analyses (Table 2). Other factors including sex, age, tumor location, preoperative CEA, histological grade, and mucinous or signet ring cell type were not significantly associated with RFS. The multivariate analysis identified a CONUT score ≥ 3 status as an independent prognostic factor associated with RFS (Table 2).

**Table 2:** Univariate and multivariate analyses of RFS in patients with colorectal cancer.

	Univariate			Multivariate		
	Hazard ratio	CI	*p*-value	Hazard ratio	CI	*p*-value
Gender(male: female)	1.004	0.613–1.614	0.986			
Age	0.597	0.186–1.911	0.3755			
Location of cancer (right side: left side)	1.06	0.630–1.740	0.8105			
Histology (tub1, tub2: others)	1.128	0.395–2.533	0.799			
Vascular invasion	3.413	1.786–7.373	0.0001	1.934	0.976–4.295	0.1079
Lymph invasion	3.372	1.995–6.003	0.0001	2.276	1.305–4.160	0.005
pT category (T1,T2:T3≦)	4.312	2.193–9.766	0.0001	2.323	1.122–5.456	0.022
N category	2.079	1.300–3.346	0.002	1.453	0.882–2.414	0.144
Preoperative CEA levels	2.218	1.380–3.550	0.001	1.773	1.095–2.874	0.022
Preoperative CA 19-9 levels	1.588	0.880–2.708	0.120			
Early complications	1.781	1.070–2.890	0.027	1.720	1.091–2.874	0.021
CONUT	2.136	1.333–3.412	0.002	1.797	1.107–2.838	0.018
PNI	2.190	1.306–3.568	0.003	1.240	0.401–1.664	0.553
mGPS	1.695	0.995–2.787	0.052			

The results of the univariate and multivariate analyses for OS are summarized in Table 3. For the univariate analyses, vascular invasion, lymph invasion, pT category, N category, preoperative CEA level, CONUT score, and PNI were significantly associated with the patients' OS. In the multivariate analyses for OS, the CONUT score and lymph invasion were independent predictive factors.

**Table 3:** Univariate and multivariate analyses of OS in patients with colorectal cancer.

	Univariate			Multivariate		
	Hazard ratio	CI	*p*-value	Hazard ratio	CI	*p*-value
Gender(male:female)	1.274	0.626–2.750	0.510			
Age	1.462	0.727–3.033	0.288			
Location of cancer (right side:left side)	1.250	0.581–2.545	0.554			
Histology (tub1, tub2:others)	1.295	0.388–8.028	0.715			
Vascular invasion	3.413	1.272–11.02	0.012	1.551	0.564–5.481	0.418
Lymph invasion	4.485	1.971–12.05	0.0002	3.146	1.339–8.641	0.007
pT category (T1,T2:T3≦)	5.300	1.874–22.18	0.0007	2.803	0.906–12.33	0.076
N category	2.417	1.201–5.017	0.013	1.536	0.735–3.321	0.256
Preoperative CEA levels	2.548	1.268–5.169	0.010	1.855	0.911–3.818	0.088
Preoperative CA 19-9 levels	1.373	0.547–3.021	0.302			
Early complications	2.023	0.950–4.125	0.067			
**Nutritional index**						
CONUT	2.092	1.123–3.873	0.035	1.853	1.257–7.921	0.018
PNI	2.316	1.076–4.715	0.033	2.121	0.883–6.481	0.139
mGPS	1.970	0.947–3.969	0.069			

### Relationships between clinicopathologic features and CONUT score status

The CONUT score was significantly associated with age (*p* = 0.002), pT category (*p* = 0.002), and early complications (*p* = 0.031) (Table 4).

**Table 4:** Clinicopathologic features of CONUT score high groups in colorectal cancer.

Variable category	CONUT score low group (n = 195)	CONUT score high group (n = 106)	*p* value
**Age (year)**			0.002
≦67	103(52.8)	37(34.9)	
> 67	92(47.2)	69(65.1)	
**Gender**			
Male	114(63.3)	66(36.7)	0.519
Female	81(66.9)	40(33.1)	
**Histology**			0.453
tub1, tub2	183(65.3)	97(34.6)	
Others	12(64.8)	9(35.2)	
**Vascular invation**			0.654
(+)	135(69.2)	76(71.7)	
(−)	60(30.7)	30(28.3)	
**Lymph invation**			0.332
(+)	96(49.2)	46(43.4)	
(−)	99(50.7)	60(56.6)	
**pT category (T1,2:T3≦)**			0.002
T1,2	74(37.9)	19(17.9)	
T3≦	121(58.1)	87(82.1)	
**N category**			0.889
(+)	123(63.1)	66(62.2)	
(−)	72(36.9)	40(37.7)	
**Early complications**			0.031
(+)	42(21.5)	35(33)	
(−)	153(78.4)	71(67)	

### Relapse-free survival and overall survival

**Figure 2:**
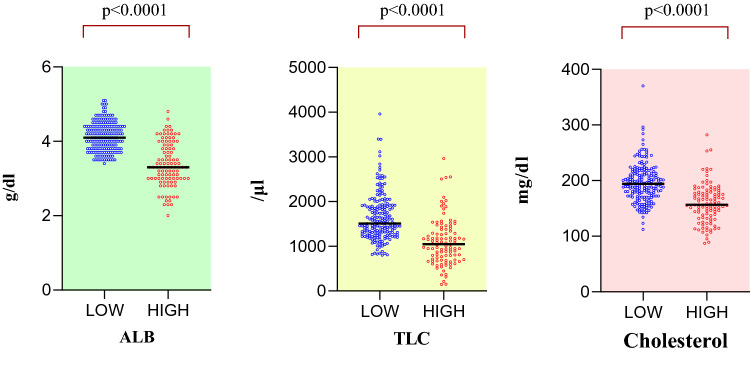
ALB, TLC, and cholesterol scattergraphs by CONUT score.

Of the total of 301 patients with a median follow-up of 1,392 days (interquartile range, 22–2,352 days) developed disease recurrence 70 (23.3%). Among the 70 patients with recurrence, liver metastases were observed in 21 (30%), lung metastases in 14 (20%), local recurrence in 11 (15.7%), para-aortic lymph nodes in 6 (8.5%), liver and lung metastases in 7 (10%), peritoneal carcinomatosis in 5 (7.1%), others in 6 (8.6%). As illustrated in Fig. 2, the low CONUT score group exhibited a significantly higher 3-year RFS rate and significantly longer OS compared to the high CONUT score group: RFS = 82.2% versus 63.3% (HR 2.14, 95%CI: 1.33–3.42, *p* = 0.002 Fig. 2), and the 3-year OS rates were 95.5% and 86.2% (HR 1.98, 95%CI: 1.27–3.83, *p* = 0.005, Fig. 2).

**Figure 3:**
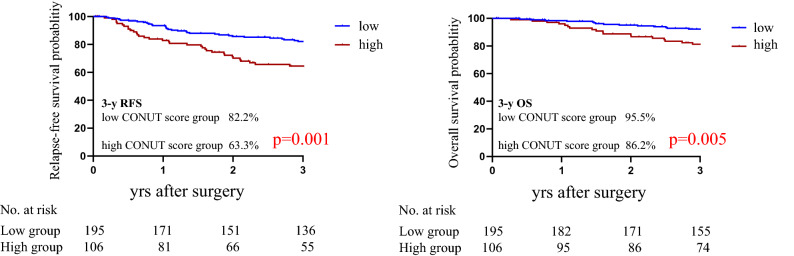
Kaplan–Meier curves of 3 years relapse-free survival (RFS) and Over all survival (OS) based on the CONUT score. High CONUT score group was significantly associated with decreased 3-year RFS and OS.

**Figure 4:**
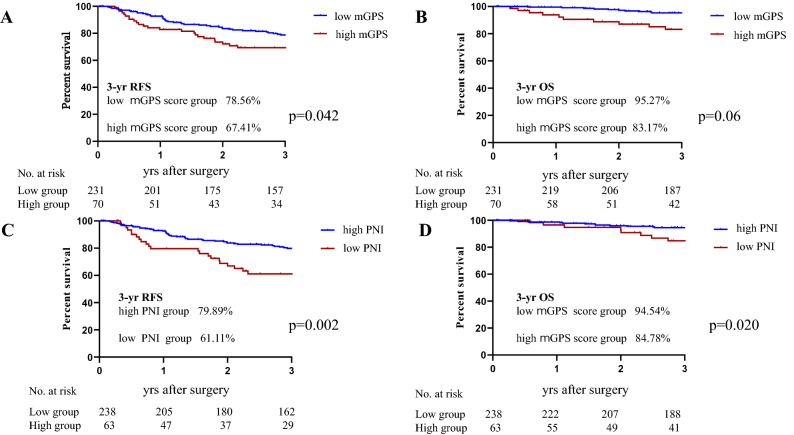
Kaplan–Meier survival curves according to (**A**) the mGPS for RFS, (**B**) the mGPS for OS, (**C**) the PNI for RFS, and (**D**) the PNI for OS.

As illustrated in Fig. 3, the 3-year RFS rate was 78.6% in the low mGPS group and 67.4% in the high mGPS group (HR 1.83, 95%CI: 1.02–3.29, *p* = 0.042), with a significant between-group difference (Fig. 4, HR 2.18, 95%CI: 1.45–4.87, *p* = 0.002). In addition, the 3-year RFS was significantly lower at 61.1% in the low PNI group compared to 79.9% in the high PNI group (HR 1.83, 95%CI: 1.02–3.29, *p* = 0.042, Fig. 3). The 3-year OS was 95.2% in the low mGPS group and 83.1% in the high mGPS group (Fig. 3, *p* = 0.06), with no significant difference between the low and high mGPS groups. However, the 3-year OS was significantly lower at 84.8% in the low PNI group compared to 94.5% in the high PNI group (HR 2.88, 95%CI: 1.18–7.04, *p* = 0.020, Fig. 3).

### AIC (Akaike's information criterion) of each index model

The AIC values of each index for RFS were 723.713 for the CONUT score, 726.948 for the PNI, 728.146 for the mGPS. According to this comparison, the CONUT score had the best goodness-of-fit, followed by the PNI and mGPS. In OS, the AIC values of each index for RFS were 315.459 for the CONUT score, 316.516 for the PNI, 318.074 for the mGPS; thus, the CONUT score had the best goodness-of-fit, followed by the PNI and mGPS.

### Correlation between albumin, lymphocyte count and CRP

The correlation between albumin concentrations and CRP and the correlation between albumin concentrations and TLC were compared. As a result, CRP was R = 0.436, TLC was R = 0.15579, and CRP was more correlated with albumin concentrations (Fig. 4).

## Discussion

We evaluated and compared the prognostic impacts of the CONUT score, the PNI, and the mGPS in 301 patients with colorectal cancer, and our analyses revealed that only the CONUT score was an independent prognostic factor for RFS and OS, and it was superior to the other nutritional-based markers in terms of predictive ability for prognosis before initial treatment.

The CONUT score is known as a nutritional assessment index. Recently, the CONUT score has been reported to help estimate postoperative recurrence rates and predict the prognosis of patients with gastrointestinal (GI) cancer. The CONUT score is composed of three common factors: the serum albumin level, which is a nutritional index, the peripheral lymphocyte count, and the total cholesterol concentration. Serum albumin has been reported to correlate with tumor necrosis because inflammatory cytokines reduce albumin synthesis^9^. As a result, hypoalbuminemia is reported to be a risk factor for the increased prevalence of high-grade colorectal cancer^10^. Peripheral lymphocytes, which play an important role in the immune response to tumors, are known to indicate an individual's immunological and nutritional status^11^. We reported that an elevated neutrophil-to-lymphocyte ratio on postoperative day 7 is a significant independent predictor of reduced RFS for patients with stage II colorectal cancer^12^. Lymphocytes, the main cells of the body's immunity, make an immune response to tumor cells, so a reduction in lymphocytes leads to a reduced ability to block tumors in the body. Therefore, high NLR levels are effective for tumor survival and lead to poor prognosis. Hypercoagulability of blood is leading for metastasis of malignant tumors^13,14^. The total cholesterol concentration is known as an indicator of a patient's reserve calories^15^. Other studies indicated that low serum cholesterol levels were associated with a poorer prognosis in patients with various cancers^16–18^. It remains unclear why low serum cholesterol levels are associated with a poor prognosis. The total cholesterol levels have been reported to correlate with tumor progression as tumor tissue reduces the body’s plasma cholesterol levels and caloric intake^19^. Therefore, hypocholesterolemia is not considered to be the cause of cancer but is thought to be caused by cancer^18,20^.

We observed herein that the CONUT score was associated with age, pT category, and early complications. Elderly patients often have poor nutritional status compared to younger people. The number of early complications after surgery may have been affected by our patients' poor nutritional status.

The PNI, which is the nutritional index calculated using the peripheral lymphocyte count and the serum albumin level, was reported to be associated with the survival of CRC patients^21^. The GPS has been used in several different oncological field and could, due to the routine assessment of albumin and CRP, be assessed in all aspects of the prospective care of patients with cancer^22,23^. In the present study, although the PNI and the CONUT score were found to be predictive factors for RFS and OS in the univariate analyses, the results of the multivariate analysis demonstrated that only the CONUT score was an independent prognostic factor for these patients with CRC.

In addition, the CONUT score more accurately predicted the survival of the CRC patients than the PNI and the mGPS. Although the CONUT score. PNI, and mGPS have common factors for nutrition, they led to different results. We thus investigated the reasons why the CONUT score was superior to the PNI and mGPS in predicting the patients' prognoses. Notably, an additional factor evaluated in the CONUT score which is not included in the mGPS or the PNI is the total cholesterol concentrations. The mGPS is composed of albumin and CRP, and PNI is composed of serum albumin concentrations and TLC. Serum albumin concentrations was included in all scores, and the correlation between albumin and TLC and CRP was lower in TLC (Fig. 4). It may be diagnosing a factor associated with recurrence that is different from serum albumin concentrations. It was considered that the diagnostic value increased because the cholesterol variable was further increased in the group separated by serum albumin concentrations and TLC.

Our study is that post operative early comlications were risk factors of recurrence. It has been reported that the risk of recurrence increases with anastomosis leakage, which is one of the postoperative complications^24^. There are also reports that perioperative blood transfusion blood and subsequent development of postoperative infectious complications may be associated with a poor prognosis^25^. In our study, 8 cases of anastomosis leakage and 7 cases of early postoperative complications accompanied by blood transfusion were considered to have influenced this.

This study has its limitations. First, this study was retrospective in design and included patients from a single institution. Secondly, patients underwent a variety of invasive surgical procedures that resulted in higher mortality and morbidity, which this study did not consider. Third, there is no consensus regarding the CONUT score cut-off value, and this makes it difficult to use the CONUT score in clinical settings. We selected the CONUT cut-off value herein by performing an ROC analysis. The CONUT score is a non-specific marker of nutrition, which implies that another systemic disease can affect the CONUT score. Our findings need further review and validation in more CRC patients.

## Conclusion

Pretreatment CONUT score in CRC patients was demonstrated to be an independent predictor of RFS and OS. It was superior to PNI and mGPS in predicting the prognosis of patients who underwent curative surgery for CRC. The cost of measuring the CONUT score is very low and can easily be obtained using laboratory data from routine clinical practice. We recommend that patients calculate their CONUT score regularly before undergoing curative surgery. This could be a useful indicator for prognosis in CRC.

## Patients and methods

### Patient selection

We enrolled 301 consecutive patients with stage I–III CRC diagnosed based on the 8th edition of the American Joint Committee on Cancer (AJCC) staging system^26^ and treated with curative resection at Teikyo University Hospital, Japan during the period 2012–2017. The surgery of all of the patients was elective. This retroprospective study was approved by the ethics of Teikyo University (Registation Number; 16-032). Informed consent was obtained from all participants, the reporting of our research is in accordance with the STROBE guidelines^27^.

The variables assessed included patient age, sex, tumor location, histological grade, TNM (tumor, nodes, metastasis) stage, personal medical history, preoperative laboratory data, early complications, and follow-up status. The CONUT score and the PNI were calculated on based on the patient's preoperative laboratory data using. Early complications included anastomotic leakage surgical site infection, colitis, ileus, urinary tract infection, respiratory infection, and dysuria.

**Figure 5:**
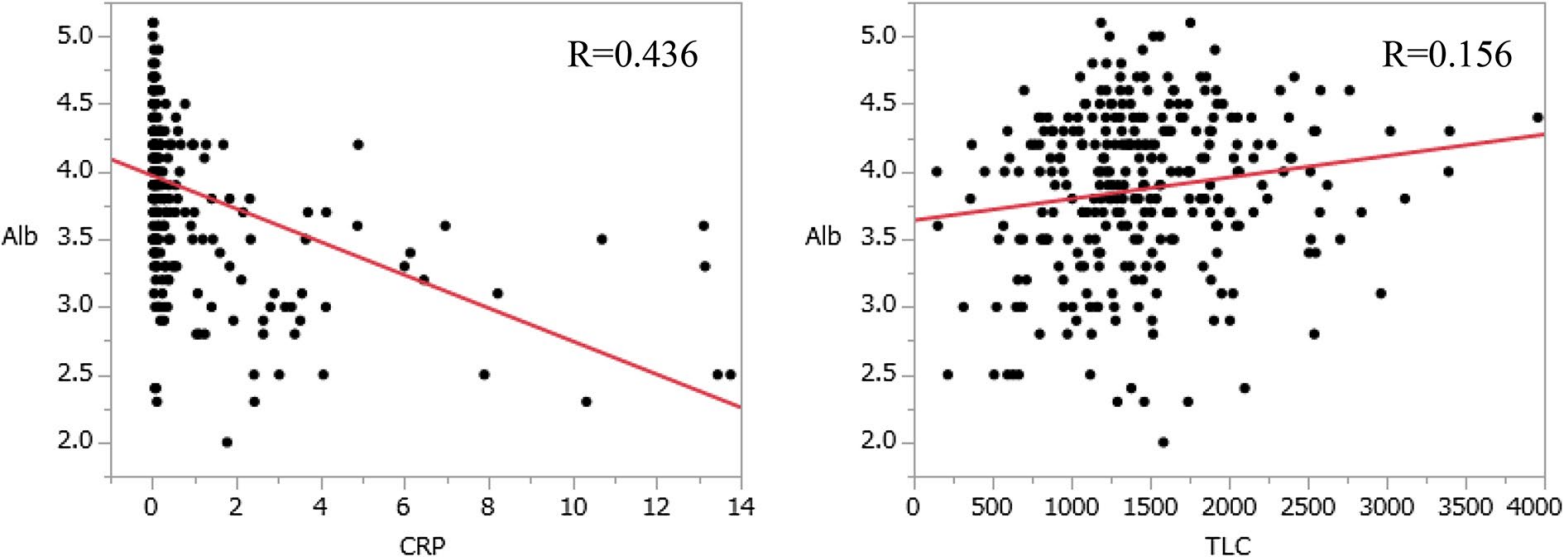
Correlation between albumin, TLC and CRP.

The following patients were excluded: (a) those who received adjuvant chemotherapy, (b) those with multiple primary malignancies , and (c) those with familial adenomatous polyposis or Lynch syndromes. Histopathological,clinical and laboratory data were obtained from the patients and blood sampling test was conducted within 1–2 weeks prior to the surgery. Total of 35 patients (neoadjuvant therapy 7, adenomatous polyposis 2, multiple primary malignancies 5 and data not available 21) were excluded (Fig. 5).

### CONUT score

Each patient's CONUT score was calculated using his/her data of serum albumin, total peripheral lymphocyte count, and total cholesterol concentrations, based on a previous report that used preoperative serum samples^28^. Albumin concentrations ≥ 3.5, 3.5 > and > 3.0, 2.99 > and ≥ 2.5, and < 2.5 g/dL were scored as 0, 2, 4, and 6 points, respectively; (2) total lymphocyte count ≥ 1,600, 1599–1,200, 1,199–800, and < 800/mm^3^ were scored as 0, 1, 2, and 3 points, respectively; and (3) total cholesterol concentrations ≥ 180, 140–179, 100–139, and < 100 mg/dL were scored as 0, 1, 2, and 3 points, respectively. The CONUT score was defined as the sum of items (1), (2), and (3). The CONUT score thus ranges from 0 to 12, with higher scores indicating a worse nutritional status.

### mGPS score

The GPS score is as follows: hypoalbuminemia (< 35 g/L) and the elevated C-reactive protein (CRP) (> 10 mg/L) was given a score of 2. Hypoalbuminemia (< 35 g/L) or CRP > 10 mg/L was score of 1. Albumin concentrations ≥ 3.5 g/dL and CRP ≤ 10 mg/L was score of 0^9^. However, hypoalbuminemia alone was not associated with reduced survival, so mGPS was created. The mGPS score 2 is hypoalbuminemia (< 35 g/L) and the elevated CRP (> 10 mg/L), score 1 was the elevated CRP (> 10 mg/L), score 0 is hypoalbuminemia (< 35 g/L) or serum albumin level (> 35 g/L) and the serum CRP lebel (< 10 mg/L)^29^.

### PNI

The PNI, which is calculated using serum albumin and the peripheral lymphocyte count, is a simple and useful score for predicting the prognosis of patients with various cancers^30^. The PNI was calculated using the following formula: PNI = serum albumin level (g/dL) + 5 × total lymphocyte count^21^. Onodera reported that this index provided an accurate, quantitative estimate of operative risk^31^. In general, resection and anastomosis of the gastrointestinal tract can be safely practiced when the index is > 45. The same procedure may be dangerous when the index is between 45 and 40. When the PNI is < 40, this surgery may be contraindicated^31^. In other studies, the cutoff value of PNI was 40, which is common, We thus set the cut-off value for the PNI as 40^21,32,33^.

### AIC (Akaike information criterion)

The AIC is a popular method for comparing the adequacy of multiple, possibly nonnested models^34^. A statistic that evaluates the predictability of a statistical model using the difference between the observed value and the theoretical value. The smaller the value, the better the fit^35^.

### Follow-up

Surgical resection was defined as radical when there was no evidence of the tumor clearance and distant metastasis was both histologically and macroscopically complete. The patients were followed up every 3 months for the first 3 years, every 6 months for the next 2 years, and once annually thereafter. At each follow-up, all of the patients underwent a physical examination as well as the measurement of serum carcinoembryonic antigen (CEA) and CA19-9 (carbohydrate antigen 19-9). They also underwent full colonoscopies at 1–2 years after the surgery (retum cancer was every 1 year after surgery). Chest-abdominal computed tomography scans were generally obtained every 6 months. Recurrence was defined as the emergence of a radiological, clinical, and/or pathological diagnosis of cancer cells locally or distant from the original position.

### Statistical analysis

Relapse-free survival (RFS) and overall survival (OS) were calculated from the date of the patient underwent surgery to that of recurrence or death, using the Kaplan–Meier method. A Cox regression analysis was performed to identify factors that are significantly associated with RFS or OS. Probability (*p*)-values ≤ 0.05 were considered significant. The Pearson product-moment correlation coefficient was used for the bivariate correlation. All statistical analyses were performed using JMP 14 software (SAS, Cary, NC, USA).

We conducted a receiver operating characteristic (ROC) curve analysis to determine the cut-off values for the CONUT score. At each value, an ROC curve was created by plotting the sensitivity and specificity of each result under study. The score closest to the point with both maximum sensitivity and specificity was chosen as the cutoff value, and the largest number of tumors was correctly classified for clinical outcome. There was a minor difference between the optimal cut-off values of RFS and those of OS, and we therefore used the cut-off values of the RFS for the OS in order to maintain consistency and prevent confusion.

The clinicopathological factors examined were as follows: sex, age, cancer location site (right side vs. left side), histology [tub 1 and tub 2 vs. others (por and pap etc.)], preoperative CONUT score, mGPS score, PNI, presence/absence of vascular invasion, presence/absence of lymph invasion pT category (T1, T2 vs. ≥ T3), pN category, tumor size, preoperative CEA level, preoperative CA19-9 level, and early complications.

### Ethical approval

The present study was conducted in accord with the Declarations of Helsinki and was approved by the Ethics Committee of the Teikyo University (approval date, 23 August 2016, Registration Number; 16–032).

## Abbreviations

AIC: Akaike's information criterion
AJCC: American Joint Committee on Cancer
CA19-9: Carbohydrate antigen 19-9
CEA: Carcinoembryonic antigen
CONUT: Controlling Nutritional Status
CRC: Colorectal cancer
CRP: C-reactive protein
mGPS: modified Glasgow Prognostic Score
OS: Overall survival
PNI: Prognostic nutritional index
RFS: Relapse-free survival
